# The impact of brain lesions on tDCS-induced electric fields

**DOI:** 10.1038/s41598-023-45905-7

**Published:** 2023-11-08

**Authors:** Carys Evans, Ainslie Johnstone, Catharina Zich, Jenny S. A. Lee, Nick S. Ward, Sven Bestmann

**Affiliations:** 1 Department for Clinical and Movement Neuroscience, UCL Queen Square Institute of Neurology, University College London, London, UK; 2 Nuffield Department of Clinical Neurosciences, FMRIB, Wellcome Centre for Integrative Neuroimaging, University of Oxford, Oxford, UK; 3 The National Hospital for Neurology and Neurosurgery, London, UK; 4 UCLP Centre for Neurorehabilitation, London, UK; 5 Wellcome Centre for Human Neuroimaging, UCL Queen Square Institute of Neurology, University College London, London, UK

**Keywords:** Neurophysiology, Neuroscience, Computational neuroscience

## Abstract

Transcranial direct current stimulation (tDCS) can enhance motor and language rehabilitation after stroke. Though brain lesions distort tDCS-induced electric field (E-field), systematic accounts remain limited. Using electric field modelling, we investigated the effect of 630 synthetic lesions on E-field magnitude in the region of interest (ROI). Models were conducted for two tDCS montages targeting either primary motor cortex (M1) or Broca’s area (BA44). *Absolute* E-field magnitude in the ROI differed by up to 42% compared to the non-lesioned brain depending on lesion size, lesion-ROI distance, and lesion conductivity value. Lesion location determined the *sign* of this difference: lesions in-line with the predominant direction of current increased E-field magnitude in the ROI, whereas lesions located in the opposite direction decreased E-field magnitude. We further explored how individualised tDCS can control lesion-induced effects on E-field. Lesions affected the individualised electrode configuration needed to maximise E-field magnitude in the ROI, but this effect was negligible when prioritising the maximisation of radial inward current. Lesions distorting tDCS-induced E-field, is likely to exacerbate inter-individual variability in E-field magnitude. Individualising electrode configuration and stimulator output can minimise lesion-induced variability but requires improved estimates of lesion conductivity. Individualised tDCS is critical to overcome E-field variability in lesioned brains.

## Introduction

Transcranial direct current stimulation (tDCS) is proposed as an economical and non-invasive method of enhancing recovery after stroke when paired with behavioural training^1–10^. TDCS may act as a ‘primer’ for therapy by producing low levels of electrical current flow through the brain that may enhance neural plasticity^5,11,12^**.** However, the effects of tDCS in stroke vary substantially across individuals^13,14^ and rarely reach clinically meaningful levels, making it difficult to adopt tDCS into routine clinical practice. Individual differences in brain and skull anatomy lead to large variations in how much current reaches the target brain regions^15,16^. This in turn leads to substantial variability in the physiological and behavioural effects of tDCS across individuals^13,14^. Brain lesions are likely to further exacerbate this variability^17^. For example, lesions can alter the conductive properties of affected tissues^18^ that determine the path of current, and this effect is unlikely to be identical in any two patients. However, systematic accounts of how lesions influence current delivered by tDCS remain limited.

The amount of current, and the path of the current through an individual's brain can be estimated using electric field modelling^19,20^. High-resolution electric field models utilise individual MRI scans to account for the complex geometry of the head and brain. From these MR images, the head is segmented into different tissue types, each with an assigned conductivity, and the volumetric anatomical images are tessellated into a 3D mesh. The voltage distribution for the resulting finite element model (FEM) is then obtained by numerically solving the Laplace equation^21^. Electric field models can be used to estimate variability across individuals^16,22^, determine the individual stimulation intensities required to generate equivalent electric fields (E-field) across participants^15^, and optimise the electrode configuration to target specific regions^22–26^. The relationship between E-field in the brain and physiological outcomes^27–31^ indicates that controlling E-field is important for determining tDCS effects. Electric field models may therefore help boost the reliability and reproducibility of tDCS effects. However, modelling normally does not account for lesioned tissue.

When models have included lesioned tissue^17,25,32–34^ conflicting results have been reported: some data suggest lesions have no greater effect on current flow than general anatomical differences^35,36^, whilst others show profound effects of the lesioned tissue on current flow^17,32,34^. Despite these efforts, the multi-faceted nature of lesion characteristics and the complexities of their influence(s) on current flow remain largely unknown. Previous studies are based on a small number of example lesions, limiting the ability to comprehensively evaluate the effect of lesion on E-field. Moreover, it is not possible to disentangle variance resulting from the presence of a lesion versus variance resulting from inter-individual differences in anatomy. Further, large inter-individual variation in lesion location and size within vascular territories^37^ exacerbates the impact on current flow.

An additional issue is that the predictions from electric field models rely on the conductivity values assigned to the lesion. Previous studies have opted to model lesions with conductivity equivalent to cerebral spinal fluid (CSF)^17,25,32,33^ but the actual conductivity of lesioned tissue is not known. Estimates obtained from various MRI techniques vary tenfold^18^, ranging from values below that of typical grey and white matter conductivity, to above the value typically assigned to CSF. Despite the wide range of conductivity values used, the effect of lesion conductivity on current flow remains unclear.

In this study, we systematically assessed the influence of lesions on tDCS-induced E-field magnitude within two regions of interest (ROIs): primary motor cortex (M1) and Broca’s area (BA44)—two regions commonly targeted by tDCS for clinical applications to facilitate recovery from movement and language deficits^10,33,38–40^. To determine general patterns or rules that govern how lesions might alter current flow we created 630 different ROI-specific lesion ‘states’ in structural MRIs, and systematically varied lesion location, its distance from the ROI, its size, and its conductivity. We compared E-field in each of these lesioned brains to their corresponding non-lesioned brain, thereby eliminating inter-individual differences in anatomy that inevitably occur as a potential confounding variable when evaluating E-field across lesion states obtained from several scans.

We further assessed how to optimise tDCS application to compensate for the impact of a lesion. Specifically, we explored the effect of individualising tDCS montages to either maximise E-field magnitude in the region of interest or maximise current directed radial inward in the cortex^26^. We also explored the effect of individualising stimulator output to maximise E-field magnitude in the ROI and reduce E-field variability between cohorts with either lesioned or non-lesioned brains. Our results emphasise the need for individualised tDCS application in populations affected by brain damage that accommodate for the effect of lesions on E-field distribution. Finally, we present a heuristic to the application of tDCS for diverse study populations that can also be related to other forms of electrical stimulation including transcranial alternating (tACS) and random noise (tRNS) stimulation.

## Methods

### Overview

Over 2500 electric field models were performed to evaluate the effect of a comprehensive variety of synthetic spherical lesions on current flow. This approach allowed for systematically quantifying of the influences of different lesion properties, whilst controlling for the otherwise inevitable effect of individual anatomy on electric fields. In other words, by building lesion states from an individual scan (i.e., 630 lesion states from one MRI) we removed variance attributed to individual differences introduced when comparing E-field across lesion states obtained from multiple scans (i.e., 630 lesion states from 630 MRIs). Two different electrode montages with utility in stroke rehabilitation research (i.e., motor and language) were modelled. For each montage, an ROI was defined (i.e., motor cortex (M1) and Broca’s area (BA44)) and synthetic lesions were created relative to each ROI. Lesions differed in their cardinal direction from the ROI, distance from the ROI, and the lesion’s size (details below). For each lesion, simulations were run using a range of conductivity values.

### Structural MRIs

Two T1-weighted 3D structural MRIs of non-lesioned brains (P01: female, 26 years; P02: female, 25 years) were used to create a total of 630 lesioned states relative to each ROI (M1 & BA44), totalling 1 non-lesioned and 1260 lesioned brains per MRI. These brains were used for all models included in the primary analyses.

All MRIs were obtained on a Siemens 3 T TIM Trio scanner with a 64-channel head coil (176 sagittal slices, matrix size 256 × 256, 1 mm isotropic resolution, TR/TE = 1900/3.96). Informed consent was obtained from all individuals. The project was approved by the UCL Research Ethics Committee (project no: 14233/001) and was conducted in accordance with the Declaration of Helsinki.

### Electric field modelling

Electric field modelling was performed using a custom version of Realistic vOlumetric Approach to Simulate Transcranial Electric Stimulation (ROAST) 2.7^20^. ROAST is a fully automated, open-source MATLAB application that produces a 3D-rendering of E-field applied to structural MRI volumes with 1 mm^3^ voxel resolution. To estimate current flow, ROAST performs segmentation of the brain image into six tissues (white matter, grey matter, CSF, bone, skin, air) via SPM12 (http://www.fil.ion.ucl.ac.uk/spm/), places virtual stimulation electrodes, performs volumetric meshing from 3D multi-domain images (via Iso2Mesh, http://iso2mesh.sourceforge.net/cgi-bin/index.cgi—^41^ to generate the finite element model (FEM), and then solves the FEM numerically using getDP FEM solver (https://getdp.info/).

We modified ROAST (hereby referred to as ‘ROAST-lesion’) to incorporate a 7th tissue type (lesion) that underwent the same processing pipeline in ROAST. Consequently, volumetric meshing and solving the FEM were performed using the 7-tissue head model. Except for the additional lesion tissue type, ROAST 2.7 default settings were used for all simulations. Briefly, meshes generated had a maximum surface element size of 5, minimum angle of surface triangle of 20, maximal distance between centre of surface bounding circle and centre of the element bounding sphere of 0.3, maximal radius-edge ratio of 3, and target maximal tetrahedral element volume of 10. Default conductivities for the six tissues were set at: white matter 0.126 S/m; grey matter 0.276 S/m; CSF 1.65 S/m; bone 0.465 S/m; skin 0.126 S/m; air 2.5e−14 S/m; gel 0.3 S/m; electrode 5.9e7 S/m. Lesion tissue conductivities were varied. Code for ROAST-lesion is available at https://github.com/ainsliej/ROAST_lesion.

### tDCS montages

**Figure 1:**
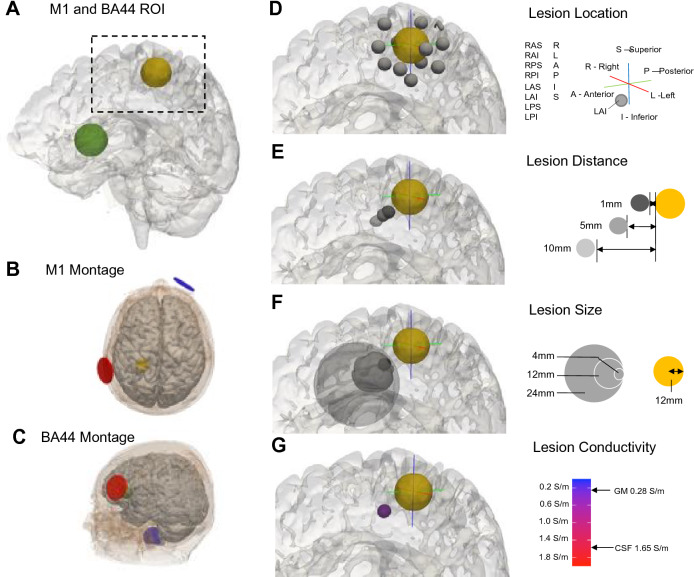
Lesion modelling approach. (**A**) The two regions of interest (ROI, M1: yellow; BA44: green) shown on an example brain. (**B,C**) tDCS montages for targeting M1 (**B**) and BA44 (**C**). Anode in red, cathode in blue. (**D**) The 14 lesion locations in relation to the location of the ROI (using M1 as an example). Lesions falling outside the brain were omitted from further analysis. (**E**) Lesion to ROI distance (1 mm, 5 mm, 10 mm), where distance was measured as the shortest Euclidean distance from the edge of the ROI to the edge of the lesion. (**F**) Lesion size (radius: 4 mm, 12 mm, 24 mm). (**G**) Lesion conductivity (0.2 S/m, 0.6 S/m, 1 S/m, 1.4 S/m, 1.8 S/m).

Two montages with utility in stroke rehabilitation research (i.e., motor and language) were selected for this study. Models simulated bipolar application of 1mA tDCS using disc electrodes (17 mm radius, 2 mm depth). In motor rehabilitation research, the left primary motor cortex (M1) is typically targeted by placing the anode over target M1 and cathode over the right supraorbital ridge^39,40^. Specifically, we placed electrodes over 10-05 coordinates CCP3 and Fp2 (Fig. 1B). In aphasia rehabilitation studies, the left frontal cortex (BA44) is often targeted by placing the anode over BA44 and cathode on the right neck (Fig. 1C). Electrodes were simulated over Exx20 and FFT7h for P01 and over Exx20 and F7h for P02.

The choice of locations used are intended to be taken as proof of principle of potential interactions between clinically used montages and lesion locations. We also note that our modelling was conducted in young adults to minimise the effect of other variables that can influence current flow, such as age-related atrophy. In reality, for some tDCS studies the exact locations of stimulation are individualised either to target intact tissue or to target anatomically or functionally defined regions^42,43^. It should be noted that, in line with Ohm’s Law, E-field magnitude values scale linearly with increases in applied current given constant conductivity.

### Lesions

A comprehensive set of synthetic lesions were positioned relative to the ROIs (Fig. 1). ROIs were 12mm radius spheres centred at manually defined M1 hand knob or BA44 (Fig. 1A). All lesions were spherical and constrained to the grey and white matter. For each ROI, a total of 630 lesion states were created from two MRIs (P01 & P02), the equivalent of 1260 lesioned brains. The advantage of this approach is that it controls for any variance introduced by differences in anatomy (e.g. skull thickness, cortical folding) which is inevitable when comparing MRI scans from healthy individuals with those from stroke survivors. Using two instead of one MRI also ensures that any-lesion induced effects on E-field magnitude are not unique to the individual anatomy of a single MRI. We here only consider lesions surrounding the target region, with the assumption that the effect of lesions further away (e.g., capsular) from the target area will generally be smaller.

Lesions were positioned relative to their cardinal direction from the ROI; either due right (R), left (L), anterior (A), posterior (P), superior (S), inferior (I), right-anterior–superior (RAS), right-anterior-inferior (RAI), right-posterior-superior (RPS), right-posterior-inferior (RPI), left-anterior–superior (LAS), left-anterior-inferior (LAI), left-posterior-superior (LPS), or left-posterior-inferior (LPI) of the ROI (see Fig. 1D). Lesions varied in distance from the ROIs (shortest Euclidean distance from the edge of ROI to the edge of lesion: 1mm, 5mm, 10mm, Fig. 1E), and in size (radii: 4mm, 12mm, 24mm, Fig. 1F). Electric field models were performed if the centre of the lesion was within the brain, and if the volume of the lesion was at least 20% of the maximum potential volume (for example, should the lesion location result in ≥ 80% of the lesion falling outside of grey and white matter, this lesion condition was excluded from the electric field model). If these conditions were met, models were run with a variety of different lesion conductivities (0.2 S/m, 0.6 S/m, 1 S/m, 1.4 S/m, 1.8 S/m) which ranged from roughly the conductivity of grey and white matter (0.28 S/m and 0.13 S/m respectively) to above that of CSF (1.65 S/m), spanning the range of values reported by McCann and colleagues^44^. Lesions were not designed to be morphologically realistic, but rather to ensure comparability and quantification of the impact on E-fields. In reality, the variation in lesion shape, location, and size that inevitably exists between individuals, along with differences in anatomy, will further exacerbate lesion-induced effects on E-field magnitude.

### Simulation outputs and independent variables

**Figure 2:**
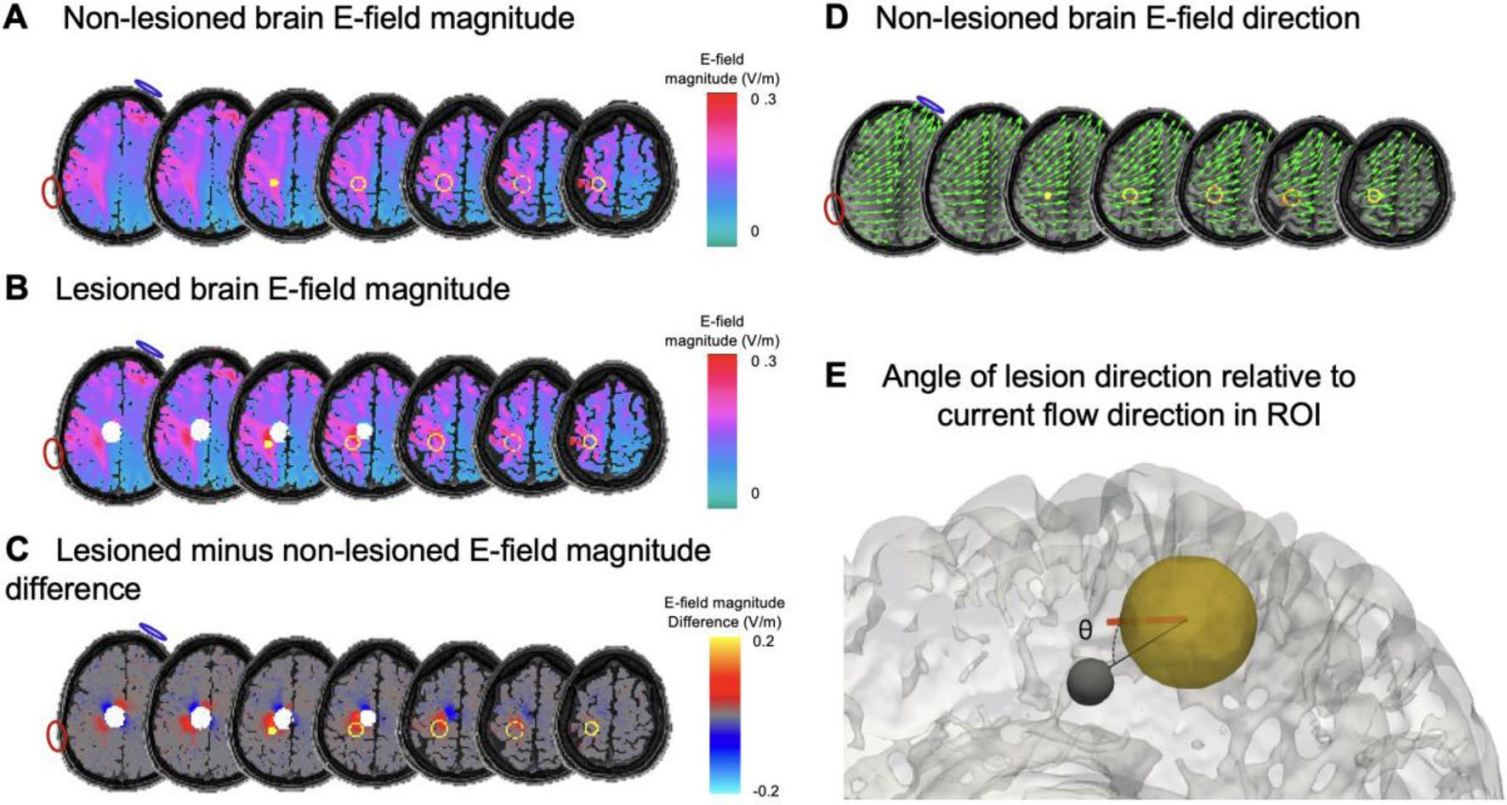
Electric field modelling in lesioned and non-lesioned brains using M1 as an example. (**A**) Electric field (E-field) magnitude image for M1 montage for the non-lesioned ‘healthy’ brain. M1 outlined in yellow; electrode locations outlined in red (anode) and blue (cathode). (**B**) E-field magnitude image for same montage but in the lesioned brain (filled white circle; RAI lesion, distance 1 mm, size 12 mm, conductivity 1.2S/m). (**C**) Difference image (lesioned minus non-lesioned brain). Note the strong increases and decreases in E-field around the lesion, with differences of approximately ± 0.1 V/m. (**D**) E-field direction in the non-lesioned brain given as vectors (green). Vector length denotes E-field magnitude (V/m). (**E**) Calculation of angle between the lesion direction (black line) and the average direction of E-field within the grey matter of the ROI (orange line).

3D images of E-field magnitude values (V/m) were extracted from the ‘healthy’ non-lesioned and lesioned brains. Differences in E-field magnitude for each lesion was calculated by subtracting the non-lesioned magnitude image from the lesioned magnitude image. The mean, the 16th percentile and the 84th percentile E-field magnitude difference were computed across the grey matter voxels within the target ROI (Fig. 2).

To calculate the direction of E-field within the ROI, the vector representing current direction (in x-, y-, and z-dimensions) was extracted from the grey matter voxels within the ROI of the non-lesioned brain. Using the mean E-field direction within the target ROI, the E-field direction vector was calculated for each lesion state in both montages.

Lesion distance, size, and conductivity were treated as continuous numerical variables in all analyses. To quantify lesion location and allow for comparison between montages, the angle between (i) the 3D E-field direction vector from within the grey matter of the ROI in the non-lesioned brain (discussed in paragraph above), and (ii) the 3D direction vector indicating the ‘movement’ from the ROI to the lesion, was calculated (see Fig. 2E). This angle (between 0° and 180°) indicates the degree to which the lesion was located in the path of current, e.g., a small angle indicates the lesion is in the path of current flow.

### Optimising tDCS

In two lesion brains, we assessed how the optimisation of the electrode montage used in individual patients can control for the impact of lesions in patient cohorts.

To this end, we used the roast-target function of ROAST v3.0^23,25,26^. We determined the optimal tDCS montage for targeting M1 in the non-lesioned brain compared to two lesioned brains created from the P01 MRI. Specifically, we used large (24 mm radius) lesions close to the ROI (1 mm distance) that were located right-anterior-inferior (RAI) and left-posterior-inferior (LPI) to the M1 ROI with a conductivity equal to CSF (1.65 S/m). The stimulator output current was set at 1mA and optimisation was constrained to a bipolar montage. For each condition roast-target optimisation was run twice with two different aims. First, the objective function was to maximise the total E-field magnitude within the grey matter of the M1 ROI. Second, we maximised specifically the component of the E-field flowing radially inward through the cortex. Both options have been discussed as viable choices for optimisation of tDCS with electric field modelling^26^. The key point of our simulations was to establish whether a lesion can, in principle, require a different optimisation solution (i.e. electrode montage), compared to a non-lesioned brain. Such a demonstration would provide important proof-of-principle that inter-individual variability in E-fields observed in a one-size-fits-all application of tDCS are exacerbated in a patient cohort.

We next demonstrate how adjusting the stimulator output can further control for the impact of brain lesions. Using the formula from Evans et al., 2020^15^ (individualised dose = (*target E-field magnitude/actual E-field magnitude)* × *fixed Dose*), the stimulator output can be either increased or decreased in order to deliver the same E-field magnitude to the target region across patients (here approximated by systematically varying lesion properties).

### Corroborating lesion-induced effects in a further three MRIs

To determine the consistency of lesion-induced effects on E-field magnitude observed in the primary analyses, we modelled a subset of 26 lesion states targeting the M1 ROI in a further three MRIs (P03: male, 26 years; P04: male, 40 years; P05: female, 23 years). Per additional MRI, a total of 1 non-lesioned and 14 lesioned states were modelled: lesions were positioned in all cardinal directions from the M1 ROI with 12mm lesion radius, 5mm distance from the ROI, and 1.4S/m conductivity (model total—P03: 8; P04: 10; P05: 8). Models were limited to fewer lesion states due to computational demands, but nevertheless qualitatively confirmed similar lesion-induced differences in E-field magnitude to that observed in the primary analyses. These results suggest that differences in E-field magnitude depending on lesion location may be generalisable (see Supplementary Figs. S1 and S2 to compare results from primary analyses to the subset of lesion states).

### Numerical stability of ROAST-lesion

Finally, we assessed the numerical stability of our simulations using the modified ROAST-lesion software. This addresses potential variability introduced by the software’s numerical stability rather than the impact of the lesion itself. Eight runs of simulations targeting M1 were conducted on the non-lesioned brain and two lesioned brains created from the P01 MRI (lesion: 24mm radius, 10mm distance, 1.4 S/m conductivity, and located either RAI or LPI to the M1 ROI). The maximum difference between E-field magnitude extracted from the grey matter of M1 was 0.0035 (V/m) across runs. Differences in E-field magnitude between conditions was around an order of magnitude higher in cases. There were no differences in stability between the conditions (see Supplementary Fig. S3).

### Analysis

All MRI manipulations were performed using tools from the FMRIB software library (FSL^45^) and all data analysis was performed using R v4.0.3 in RStudio v1.3.1093 with an alpha level cut off 0.05. Shapiro–Wilk normality tests confirmed that percentage difference in E-field magnitude results were normally distributed for all but a few conditions (size, distance, conductivity) (*p* > 0.05), therefore parametric tests were favoured.

Linear mixed effects models for each montage/ROI (M1 and BA44) separately assessed the effect of (i) lesion distance, size, conductivity on *absolute* mean E-field magnitude, (ii) lesion location on mean E-field magnitude, this time accounting for *sign*, and (iii) interactions between these variables. For all analyses, MRI identifier (i.e., P01/P02) was included as the random effect impacting intercept. Additional linear mixed effects models included both montages to confirm whether lesion effects differed across montages. Montage was included either as an additional random effect on intercept or as a fixed effect.

For tDCS optimisation analyses, we extracted and qualitatively compared E-field magnitude in the M1 ROI of three brains across the different optimisation parameters.

## Results

### Overview

**Figure 3:**
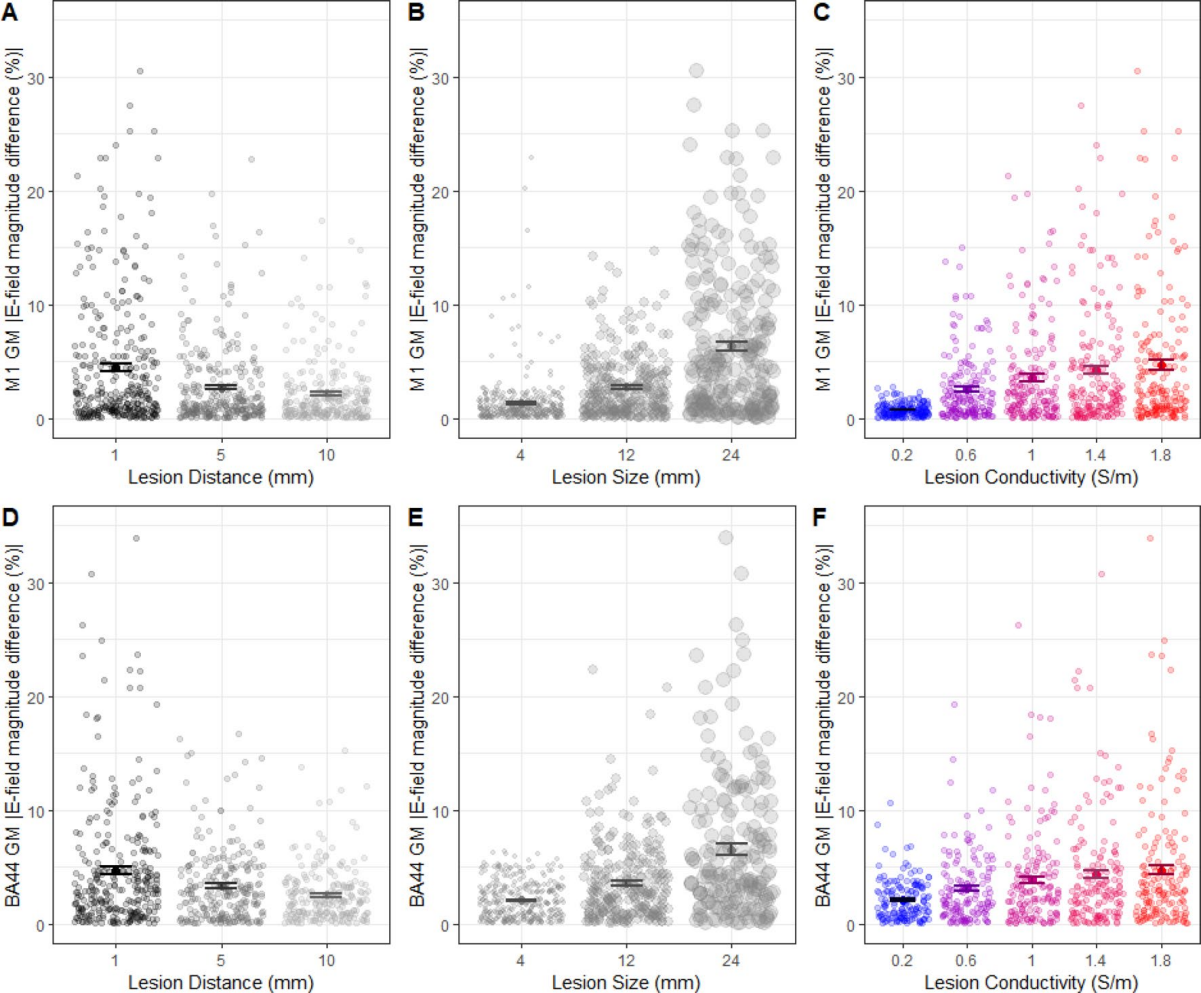
Larger lesions, which are closer to the cortical target region, and have high conductivity have a greater impact on E-field magnitude within the target area. (**A–C**) Scatter plots including the mean and standard error (SE) of the absolute percentage difference in E-field magnitude in M1 (compared to non-lesioned brain) caused by lesions with different sizes, distances, and conductivity. Data are the results from individual simulations of each lesion state. Individual data points are jittered on the x-axis for display purposes. (**D–F**) Same as in (**A–C**), for the cortical target area in BA44 GM.

**Figure 4:**
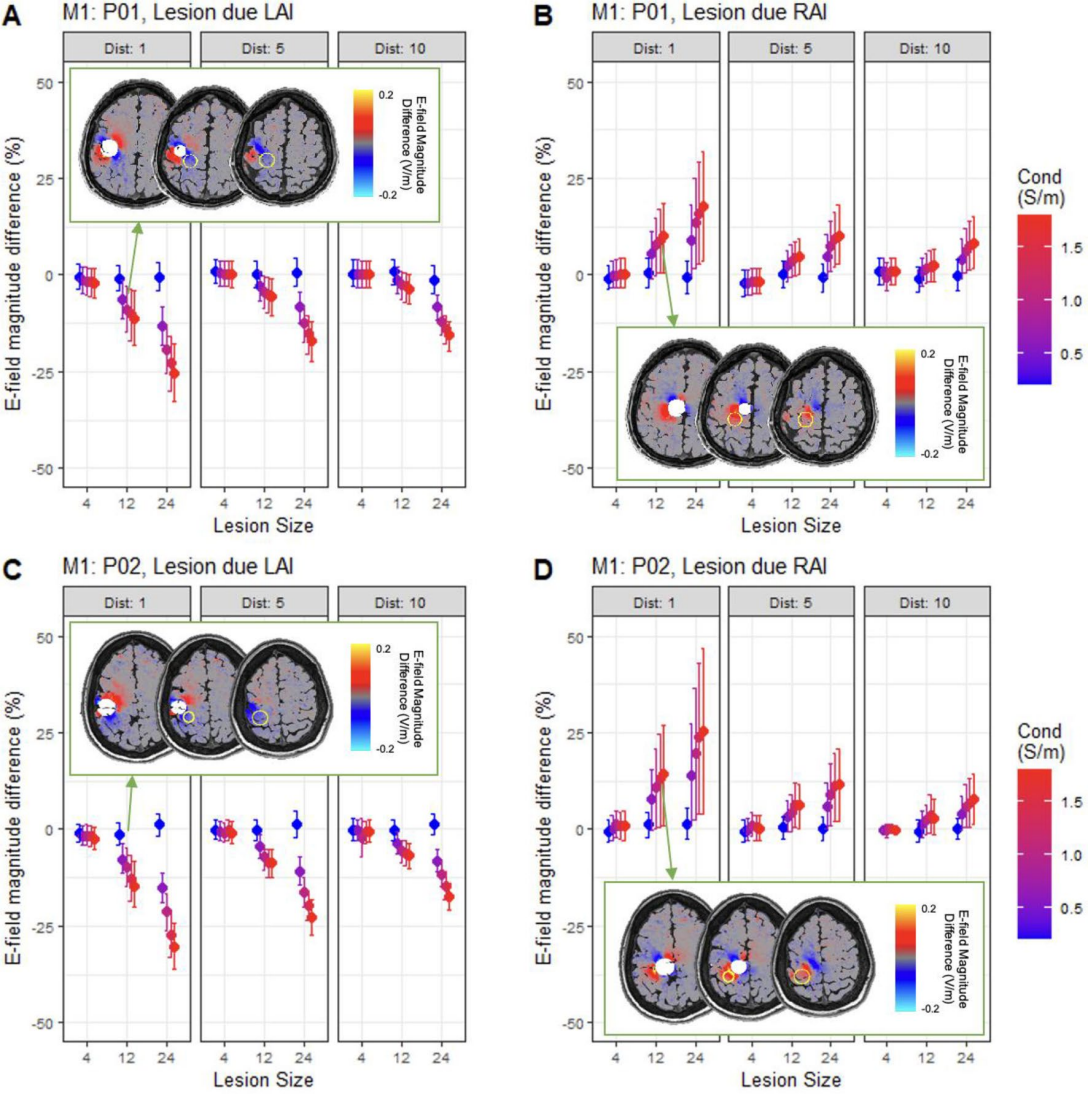
Lesion location determines the sign of E-field magnitude compared to the non-lesioned brain. Percentage difference in E-field magnitude in M1 region of interest (ROI) compared to non-lesioned brain caused by lesions due left-anterior-inferior (LAI) or right-anterior-inferior (RAI), with varying distances, sizes, and conductivities. Data points show mean percentage difference across all ROI voxels, error bars show the 16th and 84th percentile values for difference across voxels. Data for lesion states are separated by MRI identifier: P01 (A/B) and P02 (C/D). Inset: Difference in E-field magnitude across the whole brain for LAI and RAI lesions of size 12 mm, distance 1 mm and conductivity 1.4S/m (M1 outlined in yellow; lesion shown as filled white circle). Of note, cortical areas distal to the lesion show little difference in E-field magnitude (grey), whereas areas adjacent to the lesion increase (red) or decrease (blue) in magnitude by ~ 0.1 V/m. In some small regions of perilesional space, stronger magnitude differences of ~ 0.2 V/m are observed: E-field magnitude substantially decreases lateral and anterior to lesions due LAI to the ROI in (**A**) (cyan) and substantially increases lateral and posterior to lesions due RAI to the ROI in (**D**) (yellow).

**Figure 5:**
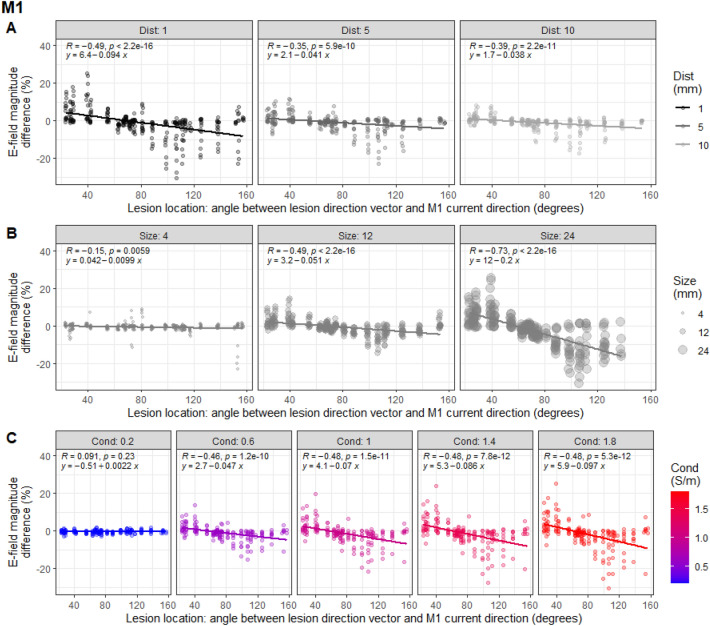
Interaction between the effect of lesion location with distance, size, and conductivity on E-field magnitude in M1. (**A**) Differences in E-field magnitude in M1 grey matter (GM) compared to non-lesioned brain plotted against lesion location, split by lesion distance (in mm). Data are the results from individual simulations of each lesion state. Lesions located in-line with the predominant orientation of current flow in M1 increased E-field magnitude, whereas those in the opposite direction caused a decrease. This was modulated by lesion distance, where closer lesions to the ROI had a greater impact on E-field magnitude. Lesion states from P03–P05 are shown in red. (**B**) Same as A but split by lesion size (radius in mm), demonstrating that larger lesions have a greater impact on E-field magnitude. (**C**) Differences in E-field magnitude plotted against lesion location, split by conductivity (in S/m), showing that lesions with higher conductivity have a greater effect on E-field magnitude. Lesion states from P03–P05 are shown in black.

**Figure 6:**
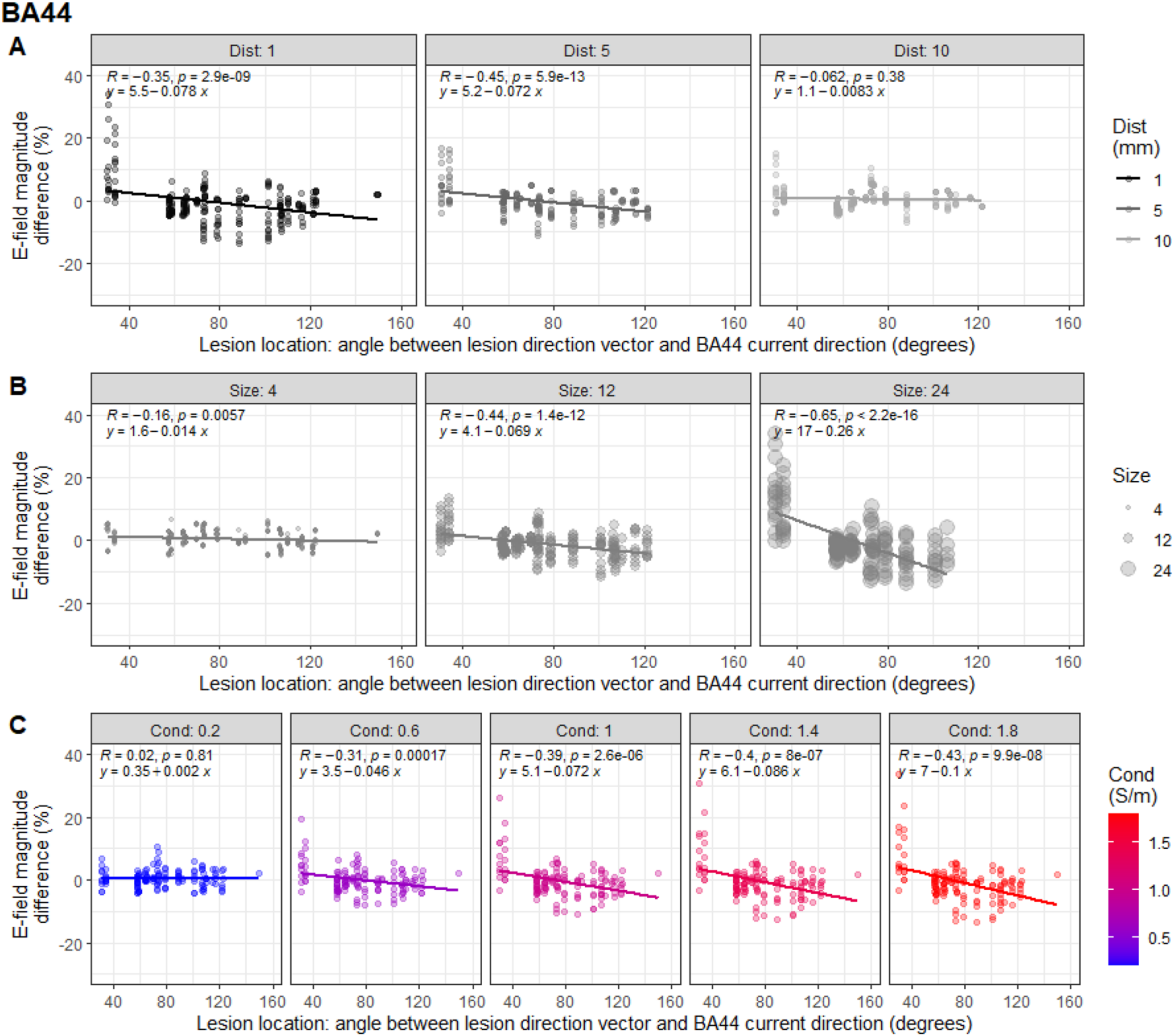
Interaction between the effect of lesion location with distance, size, and conductivity on E-field magnitude in BA44. (**A**) Differences in E-field magnitude in BA44 grey matter (GM) compared to non-lesioned brain plotted against lesion location, split by lesion distance (in mm). Data are the results from individual simulations of each lesion state. Lesions located in-line with the predominant orientation of current flow in BA44 increased E-field magnitude, whereas those in the opposite direction caused a decrease. This was modulated by lesion distance, where closer lesions to the ROI had a greater impact on E-field magnitude. (**B**) Same as (**A**) but split by lesion size (radius in mm), demonstrating that larger lesions have a greater impact on E-field magnitude. (**C**) Differences in E-field magnitude plotted against lesion location, split by conductivity (in S/m), showing that lesions with higher conductivity have a greater effect on E-field magnitude.

In the primary analyses, electric field models were conducted on 899 lesioned brains when targeting M1 (P01: 490; P02: 409). Brains of remaining lesioned states (P01: 140; P02: 221) were excluded due to meeting exclusion criteria (lesion centre was outside of the brain/ lesion volume within grey and white matter < 20% of maximal potential volume for the lesion). When targeting BA44, models from 790 lesioned brains were used (P01: 435; P02: 355; lesion states excluded: 195 and 275 respectively). Note that there are fewer simulations for the BA44 montage due to the proximity of the lesion to the cortical surface. Analyses for all lesion conditions are highlighted in Figs. 3, 4, 5 and 6. Crucially, because we used synthetic lesions, we were able to control for sources of variance in E-fields that arise from inter-individual differences in anatomy^15,16,32,46,47^.

### Larger lesions, closer to the target region, with higher conductivity have a greater impact on electrical fields within the cortical target

First, we evaluated the effects of lesion distance, size, and conductivity on the *absolute* mean E-field magnitude difference in the ROI. To this end, we used two linear mixed effect models, one for the M1 montage and one for the BA44 montage, with MRI identifier (P01/P02) as a random effect impacting intercept.

For both montages, lesions with closer distance (M1: F(1,894) = 16.9, β = −0.99, p = 4.3e−5, Fig. 3A; BA44: F(1,785) = 46.6, β = −1.05, p = 2.1e−11, Fig. 3D), larger size (M1: F(1,894) = 90.4, β = 2.31, p < 2.2e−16, Fig. 3B; BA44: F(1,785) = 178, β = 2.11, p < 2.2e−16, Fig. 3E), and higher conductivity (M1: F(1,894) = 27.4, β = 2.25, p = 1.4e−10, Fig. 3C; BA44: F(1,785) = 54.6, β = 1.63, p = 3.8e−13, Fig. 3F) had a greater impact on absolute difference in E-field magnitude within the ROI. There was no change in the significance of results if data from both montages/ROIs were included together in one linear mixed model with montage included as an additional random effect. Adding montage as a fixed effect also did not change results, and there were no significant interactions between montage and the other variables (p > 0.14).

These findings indicate that the presence of a lesion can drastically alter E-field magnitude in a cortical target compared to a non-lesioned brain, with the greatest impact (15–42% difference in E-field magnitude) observed with larger lesions, closer to the ROI, and with high lesion conductivity. These effects occur regardless of ROI location.

### Lesions in the path of current flow increase E-field magnitude, while those in the opposite direction cause E-field magnitude decreases

We then investigated the effect of lesion location on the mean E-field magnitude in the ROIs, this time accounting for *sign*. Lesions due R, RAS, and RAI of left M1 increased M1 E-field magnitude, whereas lesions due inferior, LPI, LAI, or RPI tended to decrease M1 E-field magnitude (see Fig. 4 for selected examples, and Supplementary Fig. S4 for all M1 simulations from the primary analysis). For the BA44 montage however, lesions due R and RPI increased E-field magnitude within the ROI, whereas lesions due posterior and RAS decreased E-field magnitude (see Supplementary Fig. S5 for all BA44 simulations from the primary analyses).

To quantify lesion location, and allow comparison between the two montages, the angle of lesion direction relative to current flow in the ROI was calculated. This was achieved by obtaining the angle between (i) the 3D vector describing the E-field direction within the ROI in the non-lesioned brain, and (ii) the 3D vector linking the centre of the ROI with the centre of the lesion. Angle of lesion location was a significant predictor of percentage difference in E-field magnitude in the M1 ROI, as assessed by linear mixed model (t(897) = −9.31, β = −0.06, < 2.2e−16), with MRI identifier as a random effect on intercept. The same relationship was found for the BA44 montage (t(788) =  −13.3, β = −0.07, p < 2.2e−16).

Combining data from both montages, with montage as a random effect on intercept, did not change the significance of the effect of lesion location. Including montage as a fixed effect also did not result in a significant main effect of montage, or significant interaction between montage and lesion angle (p > 0.13), again indicating that the results are consistent across the M1 and BA44 simulations.

Importantly, this indicates that lesions can cause either an increase or decrease in E-field magnitude in the ROI depending on their location relative to the path of current. Lesions more aligned with the direction of current flow in the target tend to increase mean E-field magnitude in the ROI, whereas lesions located in the opposite direction to current flow tend to decrease E-field magnitude.

### The direction of difference in E-field magnitude is modulated by lesion characteristics

Additional linear mixed models assessed whether the effect of lesion location was modulated by the other lesion characteristics. These included fixed effects of angle of lesion location relative to current flow direction in the ROI, distance, size, and conductivity, as well as interactions between the angle of lesion location and the other variables. MRI identifiers (P01/P02) were included as a random effect on intercepts. Lesion location had a greater effect for lesions with larger size, smaller distance from the ROI, and higher conductivity (see Figs. 5 and 6).

Consistent with results outlined above, all main effects were significant. Further significant interactions between lesion location and distance (M1: t(891) = 5.27, β = 0.04, p = 1.7e−7; BA44: t(782) = 6.83, β = 0.04, p = 1.7e−11), lesion location and size (M1: t(891) = −12.7, β = −0.09, p < 2.2e−16; BA44: t(782) = −13.0, β = −0.11, p < 2.2e−16), and lesion location and conductivity (M1: t(891) = −6.02, β = −0.06, p = 2.6e−9; BA44: t(782) = −9.53, β = −0.08, p < 2.2e−16) were found. Where lesions were larger, closer, and of higher conductivity, E-field magnitude differed by around 30% between lesions in-line with the predominant direction of current flow and lesions in the opposite direction to current flow.

Including all the data from the primary analyses for both montages in a single model, with montage as a random effect again did not influence the significance of the results. Furthermore, including montage as a fixed effect resulted in no significant main effect of montage and no significant interactions between montage and any of the other effects or 2-way interactions (p > 0.25). Once again, this indicates that all effects are consistent across both the M1 and BA44 simulations.

### Optimising tDCS

**Figure 7:**
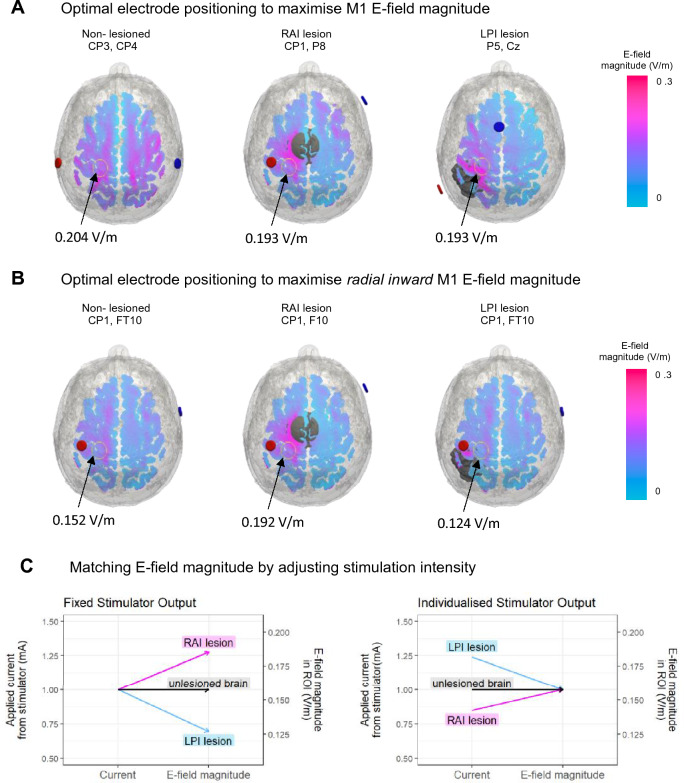
Individualising the tDCS montage and stimulation parameters. (**A**) Optimal bipolar electrode montages (anode: red; cathode: blue) that maximise the total electric field (E-field) magnitude in the grey-matter (GM) of the M1 ROI of the non-lesioned brain as well as in the brains with large (24 mm radius), close (1 mm distance) lesions due right-anterior-inferior (RAI) or left-posterior-inferior (LPI). Lesions were assigned a conductivity of 1.65S/m and tDCS was applied at 1 mA. (**B**) Optimal electrode locations that maximise radial inward current for the same cases shown in (**A**). (**C**) Demonstrating how the applied current from stimulator would need to be adjusted to achieve the same E-field magnitude within the ROI for each of the displayed lesion locations. Using formula from Evans et al., 2020: individualised dose = (target E-field magnitude/actual E-field magnitude) × fixed Dose.

We explored how the impact of lesions on E-field can be minimised by optimising tDCS and whether optimisation solutions differ for lesioned and non-lesioned brains. We first determined whether altering tDCS montage could compensate for observed lesion-induced impact on E-field magnitude. The roast-target function of ROAST v3.0 identified the optimal montage to target M1 in the non-lesioned brain compared to two lesioned brains created from the P01 MRI (lesion 1: RAI lesion; lesion 2: LPI lesion. Lesion characteristics: large (24 mm radius), close (1 mm distance), 1.65 S/m conductivity) (Fig. 7A). We then determined whether adjusting stimulator output could control for any outstanding variability in E-field magnitude.

Initial optimisation was defined as a bipolar montage that maximised E-field magnitude, across all component directions, within the M1 ROI. In the non-lesioned brain, the modelling positioned the anode on CP3 and the cathode on CP4 (CP3–CP4). This montage achieved 0.204 V/m within the ROI, compared with 0.156 V/m for the conventional, and initially used, M1 montage (CCP3-Fp2). However, for the lesioned brains, the optimal electrode locations for maximising E-field magnitude were quite different, with CP1–P8 suggested for the RAI lesion condition, and P5-Cz suggested for the LPI lesion. These montages both achieved E-field magnitudes of 0.193 V/m in the ROI, compared with around 0.182 V/m and 0.129 V/m using the original M1 montage for the equivalent RAI and LPI conditions.

While these montages increased E-field magnitude at the target location, substantially moving the electrodes will result in a change in the direction of current flow through the ROI. Previous studies indicate the importance of the direction of current flow^48–50^. Therefore we repeated our optimisation, this time based on a bipolar montage that maximises only the component of the E-field where current is moving radially into the cortical surface—thought to maximally impact the underlying neurons^50^. This resulted in more similar electrode placements between lesion conditions (Fig. 7B). For both the non-lesioned brain and the LPI condition CP1-FT10 was optimal, and for the RAI condition CP1-F10 was optimal, moving the cathode only slightly. Using this more constrained optimisation, however, resulted in a greater variation in E-field magnitude across conditions (non-lesioned: 0.152 V/m; RAI: 0.192 V/m; LPI: 0.124 V/m).

With all other factors held stable, E-field magnitude should scale linearly with the applied stimulator current. This means small adjustments to the stimulator output could be used to match E-field magnitude between conditions^15^ (see Fig. 7C). The results demonstrate that lesions exacerbate the known variability in E-field magnitude observed when applying fixed tDCS and emphasise the need for individualised stimulation protocol.

## Discussion

In this study, we systematically assessed the impact of brain lesions on tDCS-induced electric fields. We found that distance from the target region of interest, size, and conductivity of the lesion all impacted the electric field (E-field) magnitude delivered by tDCS, with increases or decreases of more than 30% (ranging from 15 to 42%) compared to a non-lesioned brain. To further probe why some lesions caused increases in E-field magnitude and others decreases, we investigated the effect of lesion location on E-field at the cortical target. We found that lesions positioned in-line with the predominant orientation of current flow in the target region increased E-field magnitude within that region. By contrast, lesions positioned maximally out-of-line with the dominant direction of current flow caused a decrease in the E-field magnitude within the target area. This effect depended on lesion distance, size, and lesion conductivity. Lesions that were larger, closer to the target, and had a higher conductivity tended to have the greatest impact—whether positive or negative—on E-field magnitude. These effects were consistent when targeting the primary motor cortex (M1) and Broca’s area (BA44) across lesion states generated from different individuals. We show that the lesion characteristics strongly influence the current delivered to an individual, an effect that likely exacerbates inter-individual variability in E-field delivered by tDCS in stroke. With E-field magnitude and current direction being primary contributors to tDCS effects, E-field variability (resulting from a lesion) likely contributes to the variable response to tDCS that has limited its clinical efficacy. Our results reveal a generalisable pattern in the influence of lesions on tDCS-induced E-field magnitude within a target region, which could be used to guide future applications for tDCS and other forms of electric stimulation including tACS and tRNS.

Previous modelling studies examining the effect of lesions on tDCS-induced E-field^17,25,32–34^ reported conflicting effects of lesions on E-field in the region of interest. For example, moderately sized lesions may add no more variance in cortical E-fields than variance attributed to general inter-individual differences in anatomy^35,36^, implying that tDCS montage and dose selection may not require extra consideration in patient populations. But lesions have also been reported to cause a decrease in E-field in the region of interest^17,34^, with larger lesions of higher assigned conductivity having the greater influence on E-field magnitude^34^. These data suggests that any lesion effects on E-field in the ROI may be ameliorated by simply increasing the dose of stimulation in a patient population. Our results confirm that lesions indeed alter E-field in the ROI and that larger lesions with higher assigned conductivity have the greatest influence on E-field magnitude. However, we present the novel finding that lesion location, with respect to the direction of current flow, determines whether E-field magnitude in the ROI increases or decreases relative to a non-lesioned brain.

A possible explanation for these directional effects is that more conductive lesion tissue draws electric current toward it, as the distribution of E-field is heavily impacted by conductivity of local tissues in the path of current. For example, wide pockets of CSF can lead to clustering of high E-field magnitudes in distinct sites^21^ or greater current shunting to deeper structures^51,52^, which is particularly evidenced in ageing populations^53^. Like our data, a recent study observed that the location of CSF pockets relative to the cortical target directly affected E-field magnitude and focality in the target region. CSF pockets located between the target region and cathode electrode, but close to the target, drew current towards the target region. This resulted in increased E-field magnitude in the target and greater focality. CSF pockets further from the target drew current closer to the reference electrode, reducing E-field magnitude and focality in the target region^54^. Predicting whether a lesion will lead to an increase or decrease in E-field magnitude, compared to the expected magnitude in a non-lesioned brain, requires consideration of both its proximity to the cortical target, but also its location relative to the path of current for a given electrode montage.

The presence of a lesion will not only alter the expected magnitude in the ROI, but likely two other important components: the focality of the E-field, and the direction of current within the ROI. We here show the complex distortions in E-field that can occur through lesions. Previous data from two stroke patients confirmed that lesions and secondary macrostructural changes such as cortical atrophy and enlarged ventricles can lead to shunting of current to stimulation “hot spots”, resulting in increased E-field magnitudes in deeper cortical structures and around the fundus of the cortical lesion^17^. We expect that the effect of lesions on E-field—including magnitude, focality, and direction of current—will depend both on the lesion characteristics themselves (i.e., lesion size, conductivity, location relative to the ROI and path of current), but also their location relative to other highly conductive tissues (e.g., ventricles), and the extent of change in other cortical structures. Because these effects will vary (sometimes substantially) in each patient, applying the same montage to every individual is likely to exacerbate variability in the effects of tDCS across patients.

By modelling synthetic lesions in the healthy brain, we were able to systematically vary several lesion parameters and determine their impact relative to each other and the ‘healthy’ non-lesioned brain. In contrast to relying on brain scans from several patients with actual lesions, this approach keeps anatomy constant across lesion states and eliminates the contribution of inter-individual differences in anatomy. It also eliminates variance introduced by the heterogeneity of the lesions themselves. This allows us to determine the true effect of lesions on E-field external to other sources of variability. To this end, we explored 630 different lesion states specific to different regions of interest, creating over 1200 unique lesion states across multiple MRIs. From these data, it is clear that individual lesions can profoundly impact the current delivered to a target region and that lesion effects on E-field—and the way lesions should be accounted for—is complex. Because individual anatomy is known to contribute to variability in E-fields delivered to a cortical target^15,16,32,46,47^ we would expect E-field variance in the presence of anatomical inter-individual variability and heterogeneous lesions to be even greater than the variance observed in either case alone.

### Implications for clinical applications of tDCS

A major issue facing the adoption of brain stimulation into clinical practice is the large inter-individual variability in responses^55^. This variability is likely to be driven, at least in part, by differences in how much current is delivered and where to^24,56^. In addition to the high degree of variability seen across healthy individuals^15,16,22^, here we show that large lesions can alter E-field magnitude by around 30%, compared to non-lesioned brains. Qualitatively, while lesion direction indeed has a greater effect for lesions with larger size, smaller distance from the ROI, and higher conductivity, the extent of difference in E-field magnitude is variable across individuals. In the present case, the synthetic nature of our lesions allowed for systematic manipulation of lesion characteristics, but the variability observed here is likely to be amplified in stroke populations with their inherently even more variable lesion characteristics.

Crucially, this data confirms that lesions have a substantial impact on E-field distribution that extends beyond expected inter-individual variance in E-field attributed to anatomical differences in the healthy brain. Therefore, assumptions generated from healthy, non-lesioned brains regarding expected E-field in an ROI cannot be generalised to the patient population. By extension, neither can expected effects of tDCS in a patient population be predicted from healthy populations, where both the structural and functional state of the brain differs^24^.

Using electric field models to individualise tDCS protocols has been suggested as a method to reduce inter-individual variability^57^ either by altering the intensity of stimulation^15^ or by altering electrode placement^26^. We used a modified version of freely available software to perform this individualised optimisation of a bipolar electrode montage for a non-lesioned brain and the two lesion conditions causing the largest increases or decreases to current flow in the M1 ROI^15,20,25^. We found that this can optimise E-field magnitude delivered to a target region but requires a bespoke electrode montage for each patient that depends on lesion characteristics. As a consequence, however, this might change the direction with which current is directed to the target location, which in itself influences the physiological effect of stimulation^22,48–50^.

We thus assessed the impact of a lesion on the direction of current in a target region. To enable comparable current direction and E-field magnitude across lesion conditions, optimisation was constrained to maximise current flowing radially inward through the cortex—thought to maximally impact the underlying neurons^22,50^. In this case, the optimal electrode montage was similar across lesion conditions, though the lesions still caused deviations of around 20% in E-field magnitude. However, as demonstrated by Evans and colleagues, stimulator output can be adjusted to match the E-field magnitude in the ROI across individuals^15^. Even for the most extreme conditions here, the maximum applied stimulator current was well within established tDCS safety guidelines^58^, making this approach a viable strategy. Taken together, lesion-induced variability can be compensated for by optimising electrode location and stimulator output in everyone based on their specific lesion characteristics.

The optimisation procedures used here are relatively time-consuming and rely on having a high-resolution whole head MRI of each patient post-stroke. While obtaining these scans is possible for some research projects, it may not be feasible for large-scale clinical practice given the availability and cost of high-field MRI. The results presented here could be used in combination with a 2D MR image, to provide some indication of whether a lesion is likely to impact the E-field magnitude in a region of interest over-and-above the normal inter-individual variability. For example, lesions that are small, distant or lie orthogonal to the path of current flow may have little impact on the E-field magnitude within the region of interest. Clinicians may also be able to anticipate how E-field magnitude may be gained or lost in the ROI by the presence of a lesion and consider whether their electrode montage should incorporate or avoid the lesion in the path of current. As a minimum, clinicians should assume that variable tDCS effects are likely exacerbated in patients with variable lesion profiles or when compared to healthy brains without lesions.

### Directions for future research

Our results also highlight that lesion conductivity can greatly impact on the E-field delivered by tDCS. In the majority of cases, lesions are modelled as CSF with a very high conductivity^17,25,32,33^. In reality, lesions are not filled with CSF and the real conductivity is therefore likely to be lower. To the authors knowledge, only one study has investigated the conductivity of lesioned tissue using magnetic resonance electrical impedance tomography (MREIT) in a single patient. Here, the predicted conductivity value was 1.2 S/m in the lesioned tissue^59^. While further research is needed to generate a robust measurement, this value is well below the 1.65 S/m typically assigned to CSF. Our results indicate that lesions with lower conductivity values have less effect, but conductivities in the region of 1.2 S/m can still result in differences in E-field magnitude of 20–25%, compared to a non-lesioned brain.

An additional complication is that the area typically segmented as a lesion is not homogenous tissue, but rather has a gradient from maximally damaged pure lesioned tissue, through partially affected perilesional tissue, to healthy tissue^60^. Rather than having a constant conductivity, it is likely that conductivity varies across the lesion. Furthermore, conductivity may not be stable across time following the stroke; diffusion MRI metrics, which are known to correlate with conductivity^61,62^, have been shown to change in the perilesional tissue from acute to chronic stages following stroke^63,64^. To ascertain the true effect of lesions on tDCS-induced current flow, further work is needed to identify accurate conductivity measures and determine whether these values change across time post stroke, location, and distance from the lesion centre.

By extension, the accuracy of E-field estimates is impacted by the exactness of the electric field models themselves. Models used here treat all tissues as isotropic, wherein fact some tissues are strongly anisotropic (e.g., white matter). This may affect the accuracy of E-field estimates, particularly in deeper brain structures^65–68^. Similarly, segmentation errors of different tissue compartments are reduced when models utilise both T1- and T2-weighted MRIs^69,70^. Nevertheless, as E-field estimates were restricted to grey matter and values were compared between non-lesioned and lesioned brains, the effect of lesion present versus absent is expected to be the same. Further, previous work suggests that accounting for white matter anisotropy did not necessarily greatly improve the predictive performance of electric field models^71^. It may also be useful to compare the explanatory power of both volumetric and boundary element methods. While more computationally demanding, surface-based boundary methods capture gyri and sulci in more detail, potentially allowing for more precise estimation of curre^1–10^.

## Conclusions

In this study, we systematically modelled the influence of synthetic lesions with different locations, sizes, and conductivities on the tDCS-induced E-field within two ROIs. A general pattern emerged across both regions. Lesions that were lying in-line with the predominant direction of current flow within the non-lesioned brain tended to increase the E-field magnitude in the ROI, while lesions that were in the opposite direction tended to decrease the E-field in the ROI. This effect was significantly modulated by lesion distance, size, and conductivity. Lesions that were closer to the ROI, larger in size and had higher conductivity induced the largest differences in E-field magnitude compared to non-lesioned brains, with increases/decreases of around 30%. These results raise the spectre of further increases in variability of current delivery in lesioned brains when no E-field modelling and individualisation of tDCS delivery is conducted. Our results also underline the need for improved estimates of conductivity in lesioned tissue to accurately estimate the true path of current flow through the brains of patients and optimise tDCS for clinical practice. Finally, we provide examples of how to individualise tDCS procedures to maximise current flow through the ROI and reduce E-field variability across individuals.

### Supplementary Information

Supplementary Figures.

## Data Availability

Code for roast-lesion, summarised data, and analysis code are available from the corresponding author on reasonable request.
