# Supplementary material for: The impact of brain lesions on tDCS-induced electric fields
Source: Sci Rep. 2023 Nov 8;13:19430. doi: 10.1038/s41598-023-45905-7 (PMC10632455; doi:10.1038/s41598-023-45905-7)
Supplement: Supplementary file 1 — Supplementary Figures. [file 41598_2023_45905_MOESM1_ESM.docx]

# Supplementary Figures


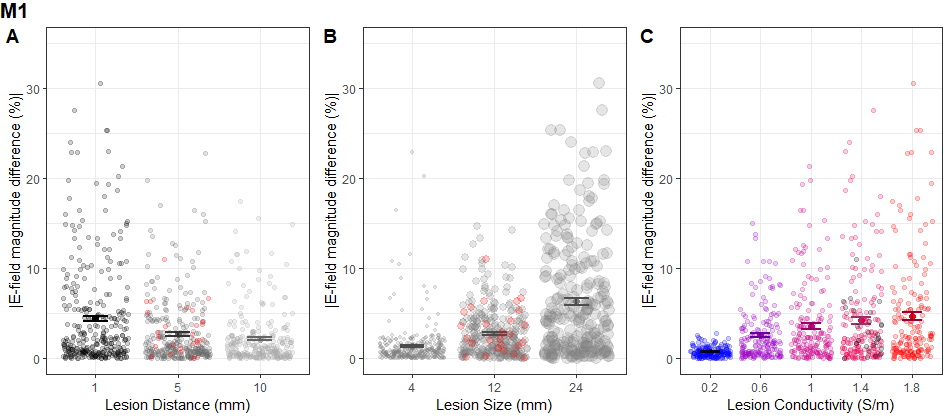


***Supplementary Figure S1 (P01-P05): Larger lesions, which are closer to the cortical target region, and have high conductivity have a greater impact on E-field magnitude within the target area***

A-C. Scatter plots including the mean and standard error (SE) of the absolute percentage difference in E-field magnitude in M1 (compared to non-lesioned brain) caused by lesions with different sizes, distances, and conductivity. Data are the results from individual simulations of each lesion state from the primary analysis and a subset of 26 lesion states. A-C show data from additional models of a subset of lesion states for P03-P05 in red (A/B) and black (C). Individual data points are jittered on the x-axis for display purposes.


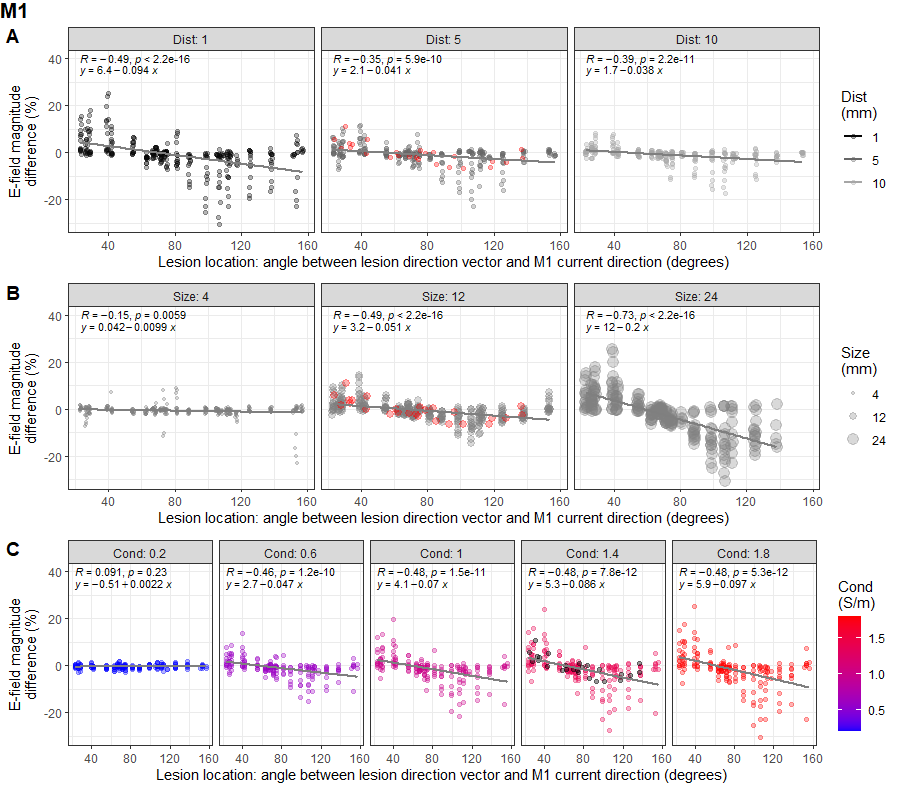


***Supplementary Figure S2 (P01-P05): Interaction between the effect of lesion location with distance, size, and conductivity on E-field magnitude in M1***

*A. Differences in E-field magnitude in M1 grey matter (GM) compared to non-lesioned brain plotted against lesion location, split by lesion distance (in mm). Data from the primary analysis (P01 & P02 in grey) and a subset of 26 lesion states (P03-P05 in red) are displayed and pooled together for analysis. Lesions located in-line with the predominant orientation of current flow in M1 increased E-field magnitude, whereas those in the opposite direction caused a decrease. This was modulated by lesion distance, where closer lesions to the ROI had a greater impact on E-field magnitude change.*

*B. Same as A but split by lesion size (radius in mm), demonstrating larger lesions have a greater impact on E-field magnitude (P01 & P02 in grey; P03-P05 in red).*

*C. Differences in E-field magnitude plotted against lesion location, split by conductivity (in S/m), showing that lesions with higher conductivity have a greater effect on E-field magnitude (P01 & P02 in colour; P03-P05 in black).*


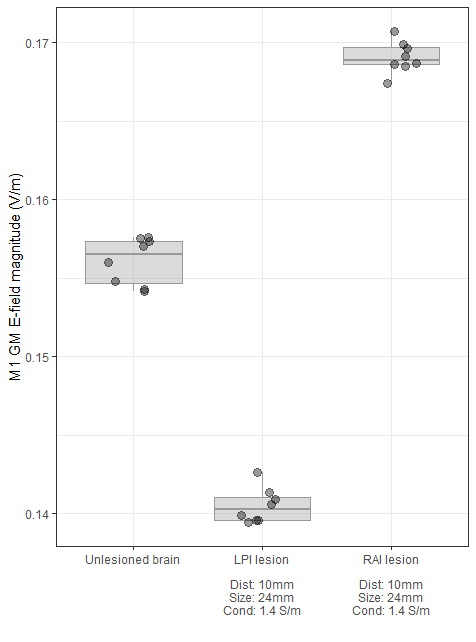


***Supplementary Figure S3: Numerical stability of E-field magnitude values across multiple simulation runs.***

*Results of 8 simulations were relatively stable, with a maximum difference of 0.0035V/m between runs. Simulations were conducted for P01 on the M1 ROI for three conditions (non-lesioned, LPI lesion, and RAI lesion) with the following lesion characteristics: size 24mm radius, 10mm distance from ROI, and conductivity 1.4 S/m.*


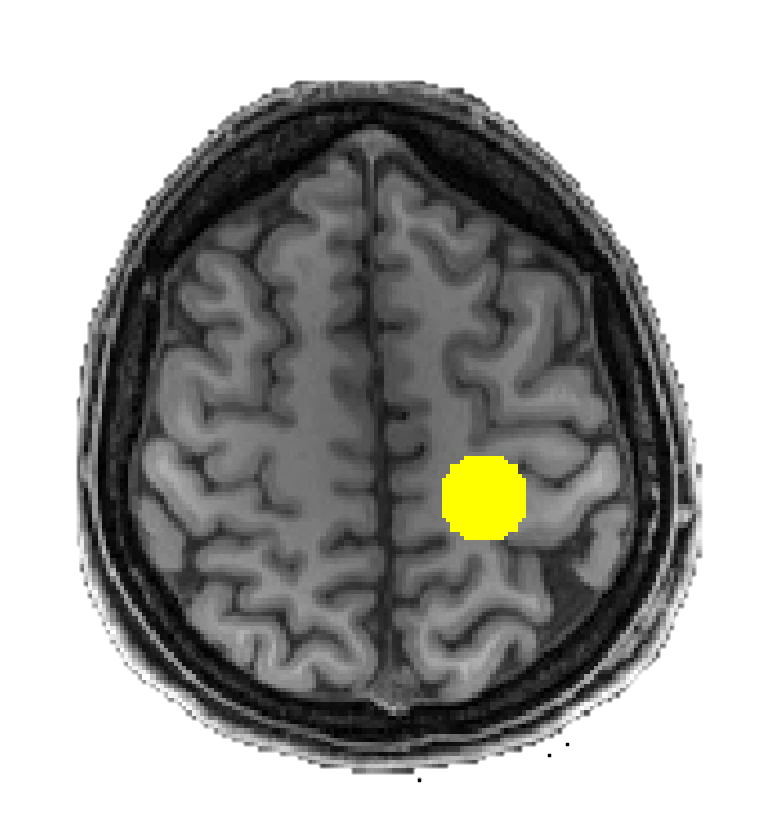

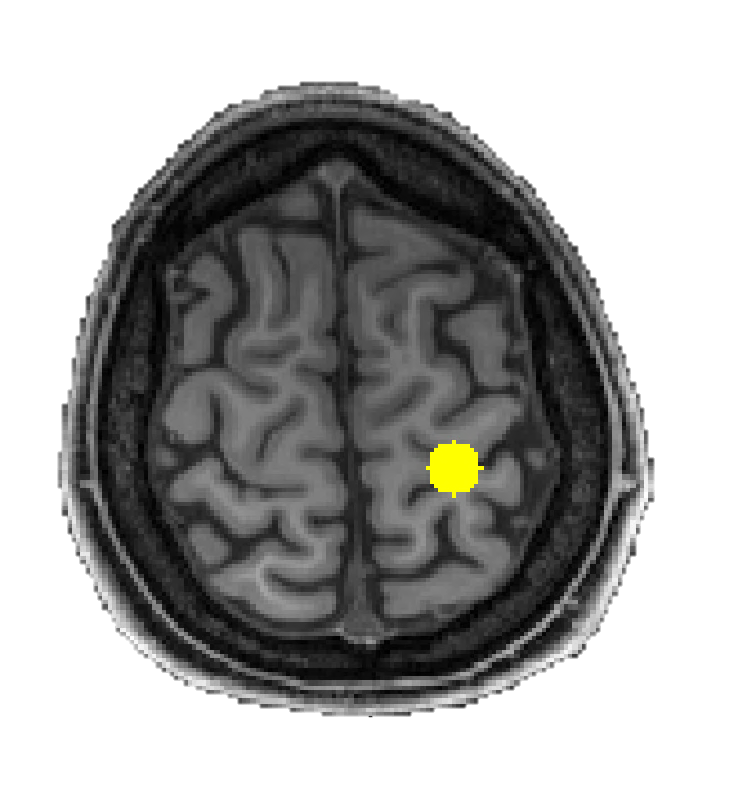

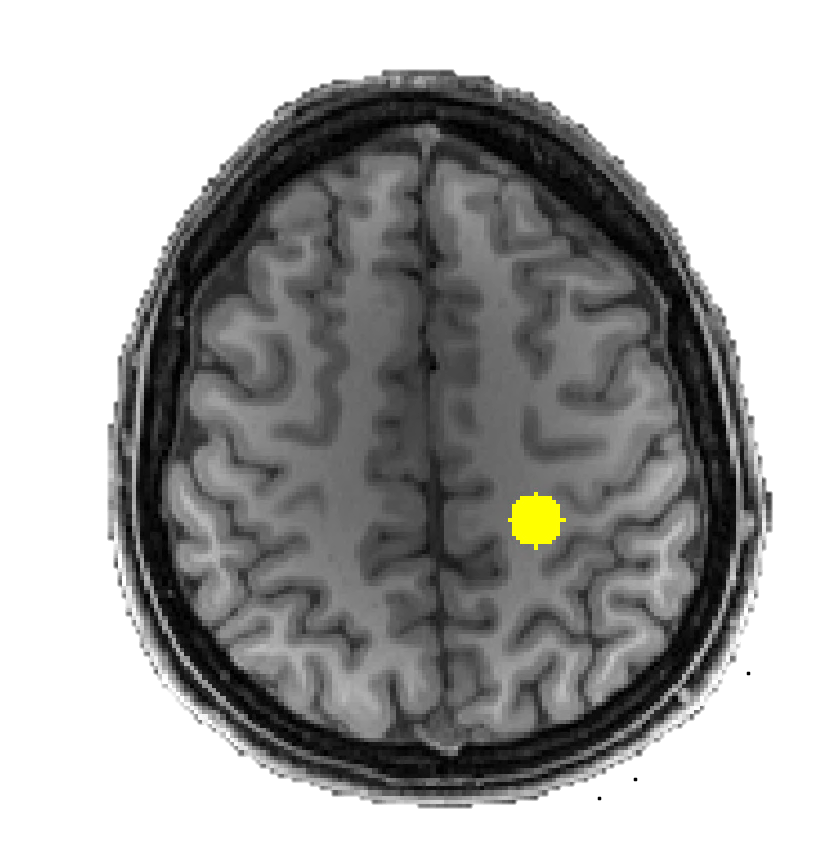

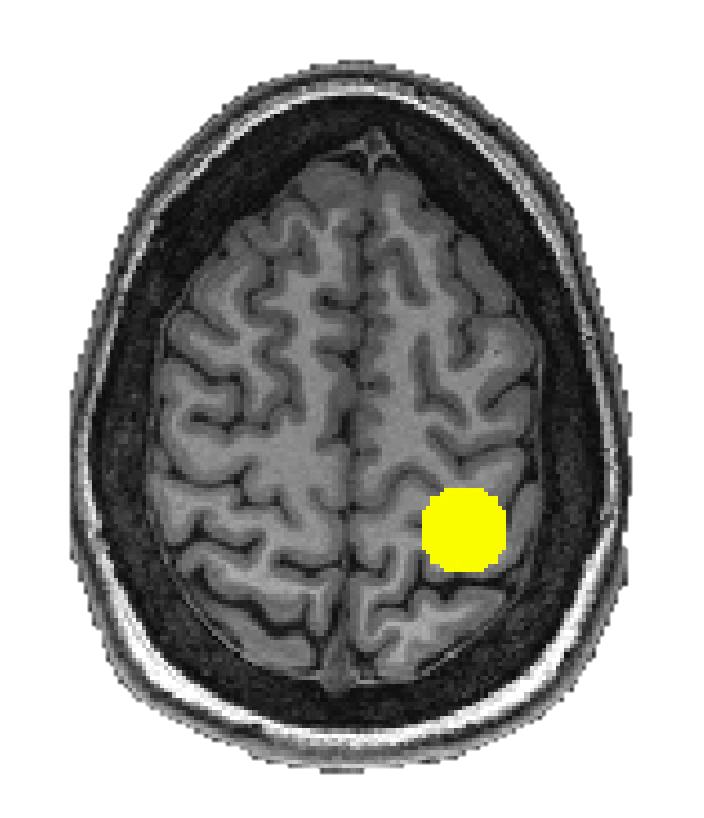

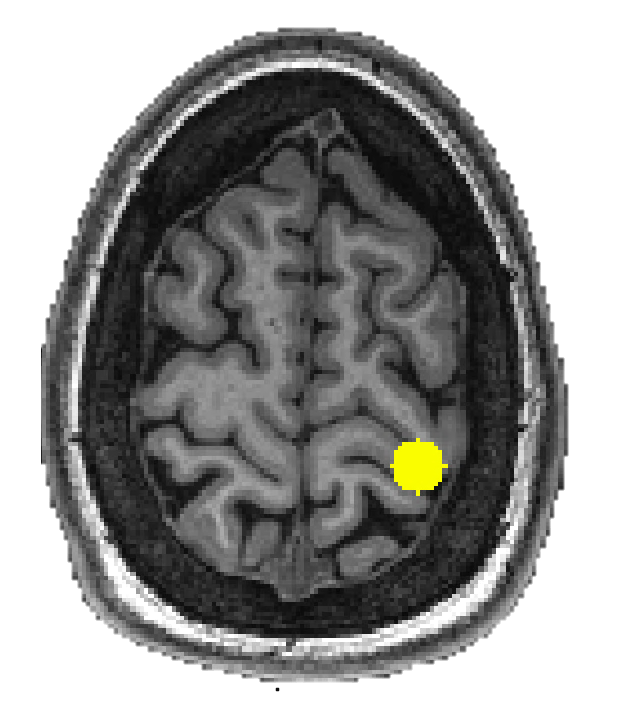

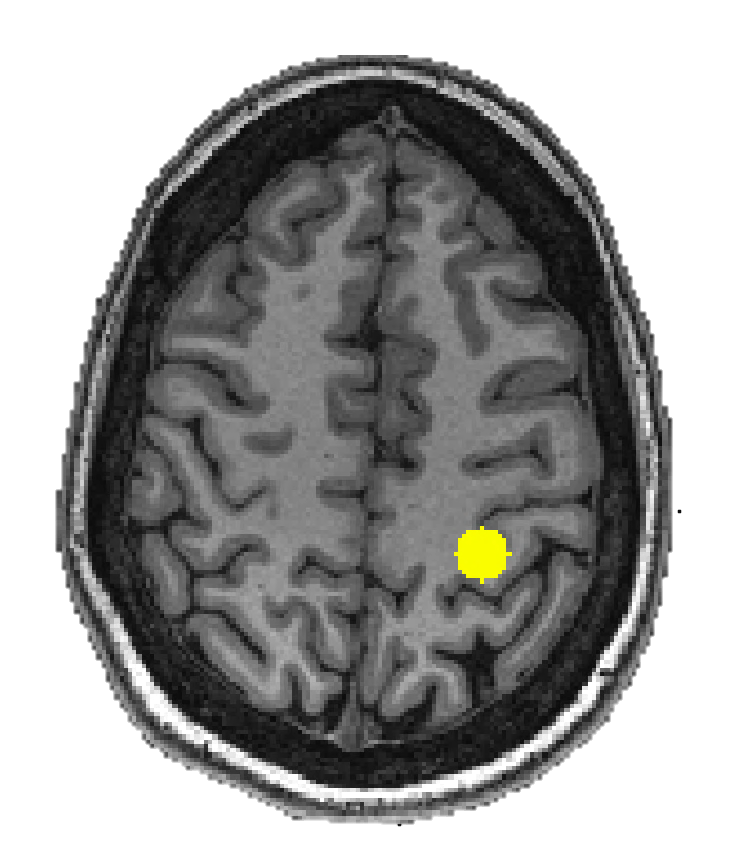


***Supplementary Figure S4: Percentage difference in M1 E-field magnitude for all lesions***

*A. Percentage difference in E-field magnitude compared to non-lesioned brain for all lesion locations, sizes (in mm), distances (in mm), and conductivities (in S/m) for participant P01.*

*B. Same for participant P02.*


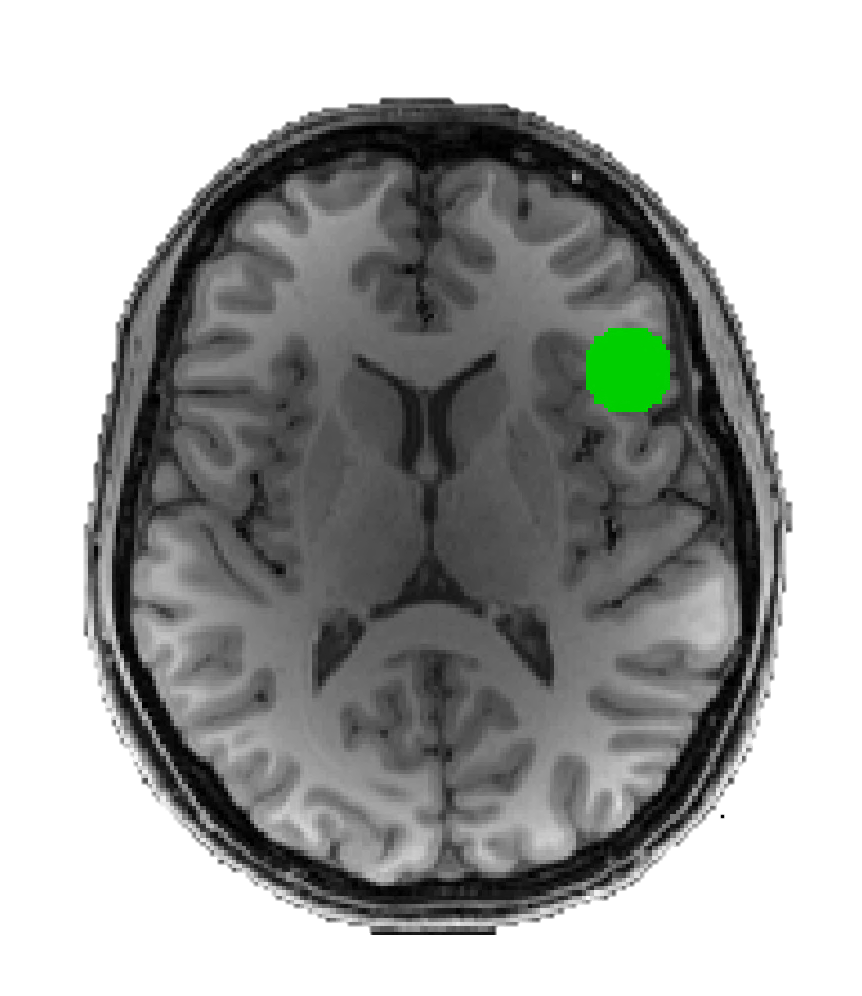

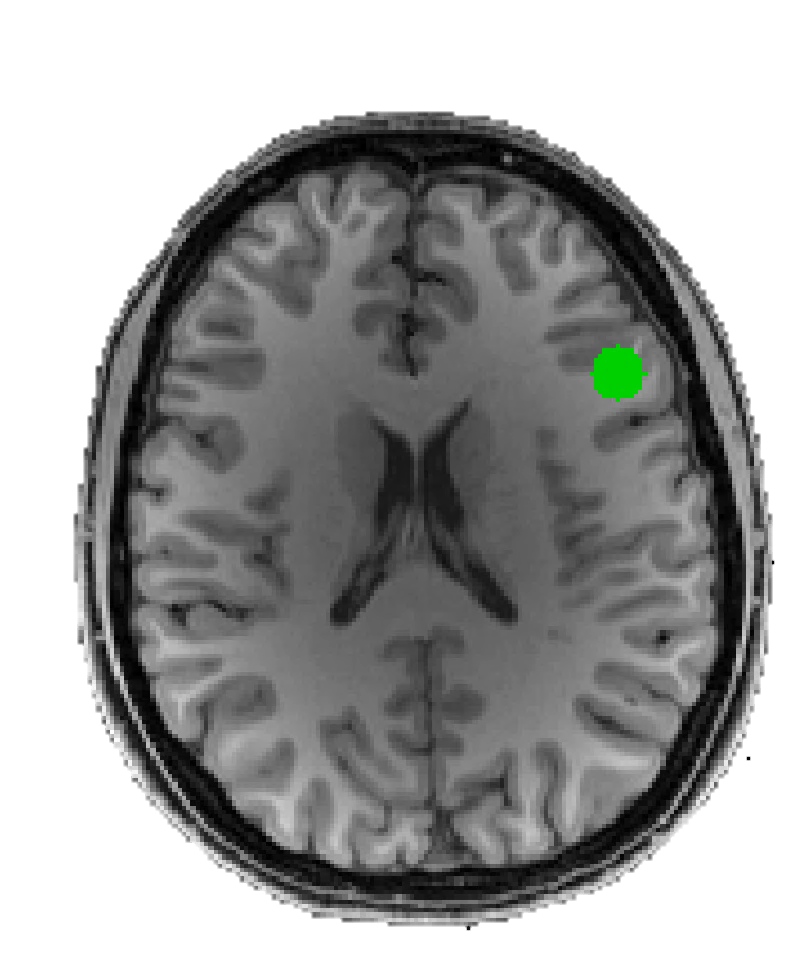

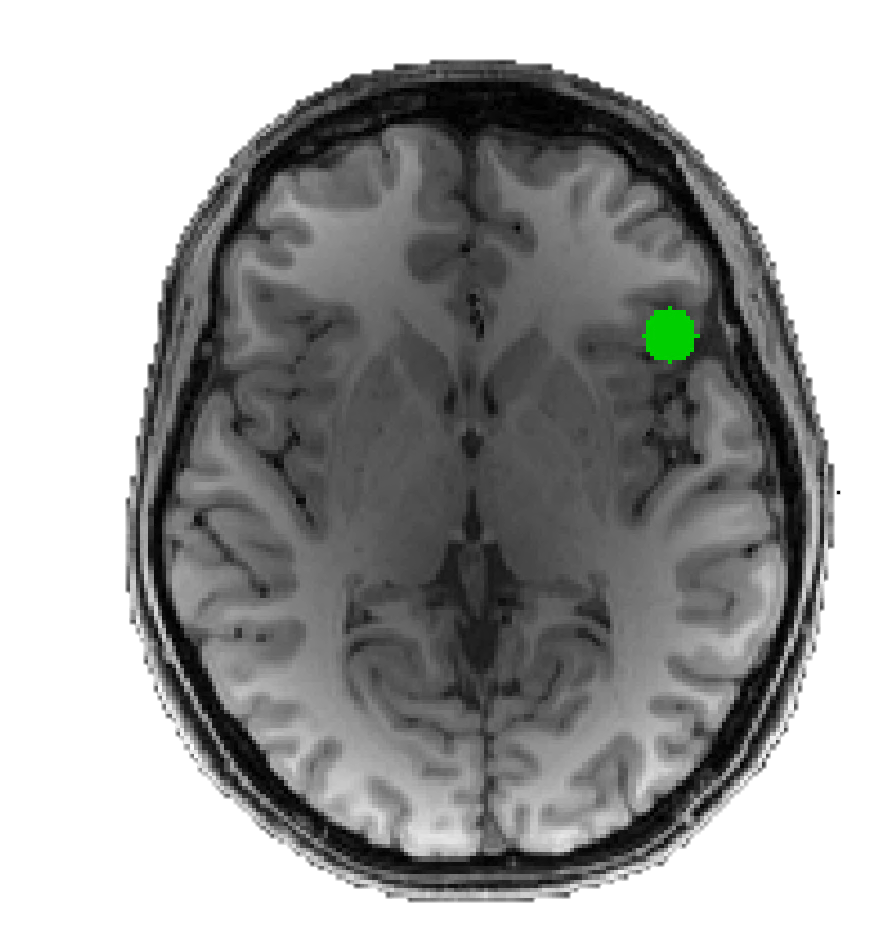

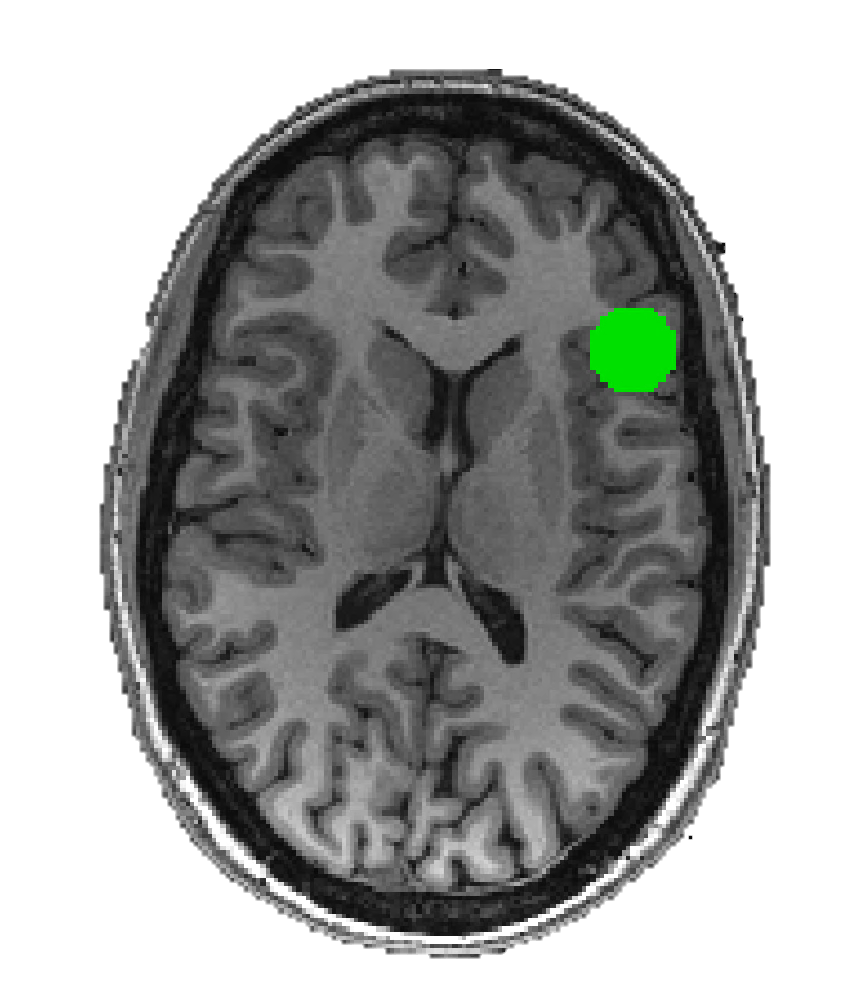

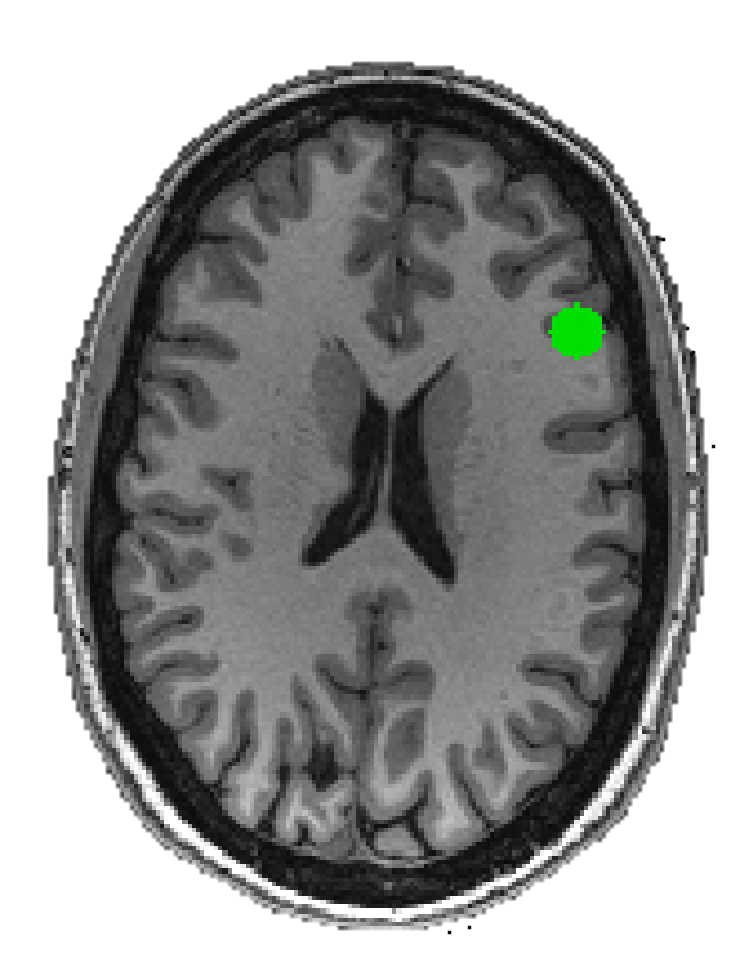

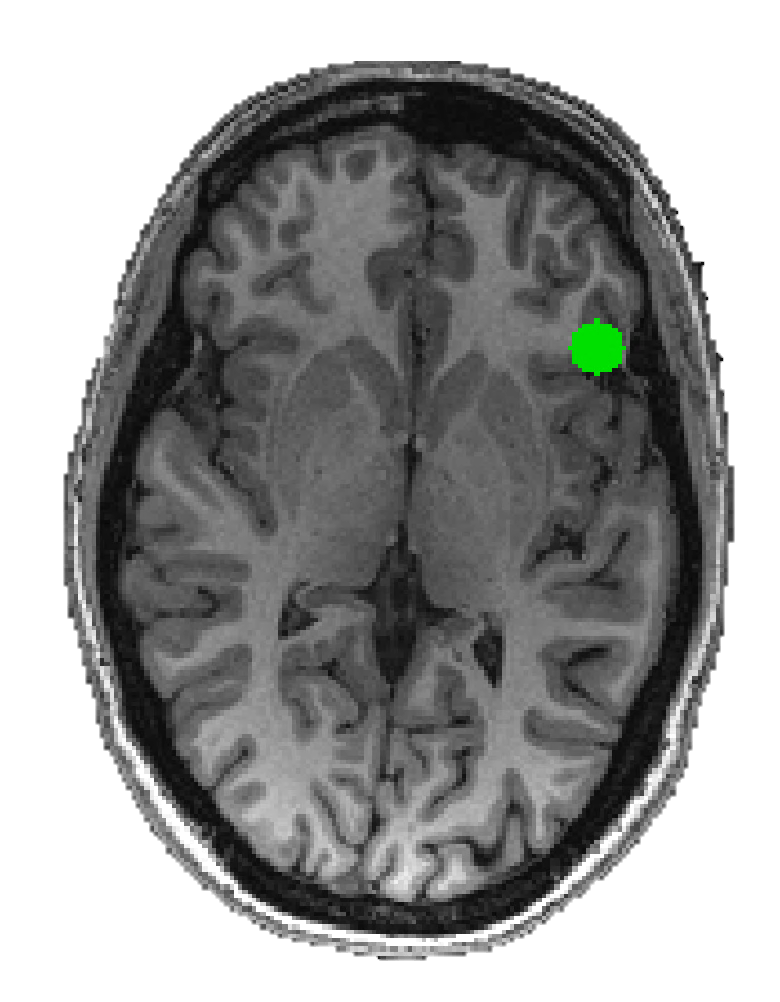


***Supplementary Figure S5: Percentage difference in BA44 E-field magnitude for all lesions***

A. Percentage difference in E-field magnitude compared to non-lesioned brain for all lesion locations, sizes (in mm), distances (in mm), and conductivities (in S/m) for participant P01.

B. Same for participant P02
